# High Efficiency Gas Permeability Membranes from Ethyl Cellulose Grafted with Ionic Liquids

**DOI:** 10.3390/polym11111900

**Published:** 2019-11-18

**Authors:** Jingyu Xu, Hongge Jia, Nan Yang, Qingji Wang, Guoxing Yang, Mingyu Zhang, Shuangping Xu, Yu Zang, Liqun Ma, Pengfei Jiang, Hailiang Zhou, Honghan Wang

**Affiliations:** 1 College of Materials Science and Engineering, Heilongjiang Province Key Laboratory of Polymeric Composition, College of Architecture and Civil Engineering, Qiqihar University, Wenhua Street 42, Qiqihar 161006, China; xjy951011@163.com (J.X.); zhangmingyuno1@163.com (M.Z.); xshp_1979_1999@163.com (S.X.); zangyu.25@163.com (Y.Z.); maliqun6166@163.com (L.M.); jpf848185@163.com (P.J.); zhouhailiang95@163.com (H.Z.); wanghonghan0628@163.com (H.W.); 2 Daqing Oilfield Construction Design and Research Institute, XiLing Road 32, Daqing 1637241, China; wangqingji@petrochina.com.cn; 3 Daqing Petrochemical Research Center, Petrochemical Research Institute, China National Petroleum Corporation, Chengxiang Road 2, Daqing 163714, China; ygx459@petrochina.com.cn

**Keywords:** ethyl cellulose, liquid, imidazole, membrane, gas permeation properties

## Abstract

Ethyl cellulose was grafted with ionic liquids in optimal yields (62.5–64.1%) and grafting degrees (5.93–7.90%) by the esterification of the hydroxyl groups in ethyl cellulose with the carboxyl groups in ionic liquids. In IR spectra of the ethyl cellulose derivatives exhibited C=O bond stretching vibration peaks at 1760 or 1740 cm^−1^, confirming the formation of the ester groups and furnishing the evidence of the successful grafting of ethyl cellulose with ionic liquids. The ethyl cellulose grafted with ionic liquids could be formed into membranes by using the casting solution method. The resulting membranes exhibited good membrane forming ability and mechanical properties. The EC grafted with ionic liquids-based membranes demonstrated *P*CO_2_/*P*CH_4_ separation factors of up to 18.8, whereas the *P*CO_2_/*P*CH_4_ separation factor of 9.0 was obtained for pure EC membrane (both for CO_2_/CH_4_ mixture gas). The membranes also demonstrated an excellent gas permeability coefficient *P*CO_2_, up to 199 Barrer, which was higher than pure EC (*P*CO_2_ = 46.8 Barrer). Therefore, it can be concluded that the ionic liquids with imidazole groups are immensely useful for improving the gas separation performances of EC membranes.

## 1. Introduction

The separation membrane represents the core of the separation technology, and the performance of the separation membrane depends largely on the membrane material and formation process. Therefore, a good gas separation membrane material must have optimal gas permeability coefficient, separation factor, chemical stability, mechanical strength and film forming ability. The membrane materials currently used in the field of gas separation are mainly organic polymer materials, including poly(dimethyl siloxane) (PDMS) [1], polysulfone (PSF) [2], poly(vinylidene fluoride) (PVDF) [3], polyimide (PI) [4], polyetherimide (PEI) [5], cellulose acetate (CA) [6], ethyl cellulose (EC) [7], etc.

EC is a functional, non-ionic cellulose ether obtained by reacting cellulose with CH_3_CH_2_Cl [8]. EC is renewable and abundantly available. In addition, EC also has unique properties otherwise not possessed by other cellulose ethers, such as chemical stability, film forming ability and good mechanical properties. The hydroxyl groups in EC are replaced by a large number of ethyl groups, forming a typical hydrophobic cellulose. The unsubstituted hydroxyl groups are connected by hydrogen bonds to form a tight coral-like network structure, which gives EC excellent mechanical properties [9,10,11]. The non-toxic, hydrophobic, mechanical, thermoplastic and film-forming properties of EC have applications in many fields, such as food, microencapsulation, filtration and medicine [9,12]. EC has also been used as a separation membrane material [13].

As early as the 1990s, literature studies reported the preparation of ethyl cellulose membranes for separating oxygen and nitrogen [14]. S.M. Chen et al. studied the gas permeability of homogeneous [15] and porous EC membranes [16] by structural modification and solvent optimization. The O_2_/N_2_ separation factors of the homogeneous membrane reached 6.2, indicating that the EC membranes have high selectivity for O_2_ [15]. Although the pores supporting EC membranes are currently for oxygen/nitrogen separation, having higher gas permeability, a lower selectivity for CO_2_/N_2_ and CO_2_/CH_4_ is generally observed [17]. In order to expand the further application of ethyl cellulose, modified EC films have also been prepared [18,19,20,21,22,23].

In another study, silyl ethers of ethyl cellulose were synthesized by the reaction of various chlorosilanes with the residual hydroxy groups of ethyl cellulose [19]. The *P*CO_2_/*P*N_2_ permselectivity values of the polymers were observed to be in the range of 15–19. The increased diffusion coefficients resulting from the introduction of silyl moiety in ethyl cellulose were observed for gas permeability; thus, their good separation performance for CO_2_ /N_2_ and CO_2_ /CH_4_ was discerned [19]. X.G. Li et al. prepared binary blend membranes of EC and poly (4-vinylpyridine) using a solution casting technique with chloroform as the solvent [20]. Remarkable and continuously enhanced selectivity was achieved for important gas pairs, including oxygen/nitrogen, carbon dioxide/methane and hydrogen/nitrogen with increasing poly (4-vinylpyridine) content [20]. The blends had higher gas separation factors and comparable gas permeabilities. Such behavior can be envisaged for all microscopically immiscible polymer blends and has important practical implications [20]. The membrane blend of EC and maleic anhydride end-capped poly(propylene carbonate) was reported to be difficult for actual gas separation due to unstable gas separation performance [21]. Q. Hu [22] and M. Moaddeb [23] also introduced nano-scale TiO_2_ or SiO_2_ particles into the EC membrane materials, which improved the gas selectivity of the membranes, but the improvement effect on gas permeability was not obvious.

Ionic liquids (IL) refer to a class of ionic compounds consisting of organic cations and organic (inorganic) anions at room temperature or near room temperature [24]. The current applications of ionic liquids in gas membrane technology are mainly reflected in the separation of CO_2_, which is mainly due to the excellent solubility and selectivity of ionic liquids towards CO_2_, especially in the case of ionic liquids containing functional imidazole groups [25]. Nikolaeva et al. [26]. have synthesized a new cellulose-derived poly(ionic liquid) (PIL) and characterized it for CO_2_ separation. The ideal CO_2_/N_2_ adsorption selectivity of poly(diallyldimethyl ammonium)-bis-(triflfluoromethylsulfonyl) imide (P (CA) (Tf_2_N)) was constantly below 10 bar. The mixed gas permeation test showed that the P[CA][Tf_2_N] based film with a 5 μm thick selection layer had twice the CO_2_ flux of the conventional cellulose acetate (CA). These results indicate that the modified CA with IL is a successful method to increase permeating flow and improve process stability over a higher CO_2_ /N_2_ and CO_2_/CH_4_ gas mixture concentration and pressure range.

In this study, membranes from ethyl cellulose grafted with ionic liquids with imidazole groups with high gas permeabilities and selectivities were prepared. An in-depth study of the relationship between the membrane structure and CO_2_ separation performance was studied subsequently.

## 2. Experimental Section

### 2.1. Materials

**Scheme 1:**
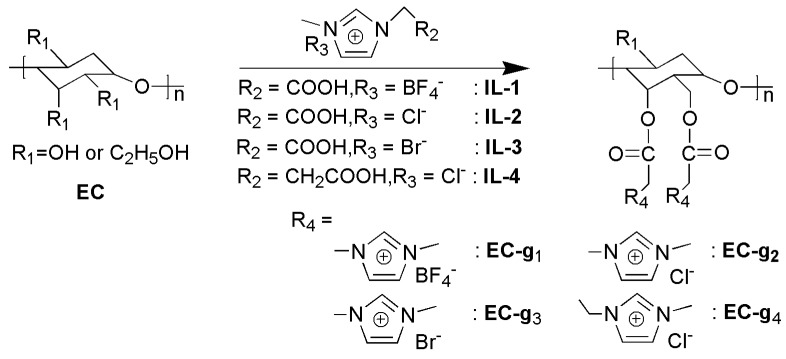
Synthetic route for obtaining ethyl cellulose (EC) grafted with ionic liquids (EC-g1–EC-g4).

**Scheme 2:**
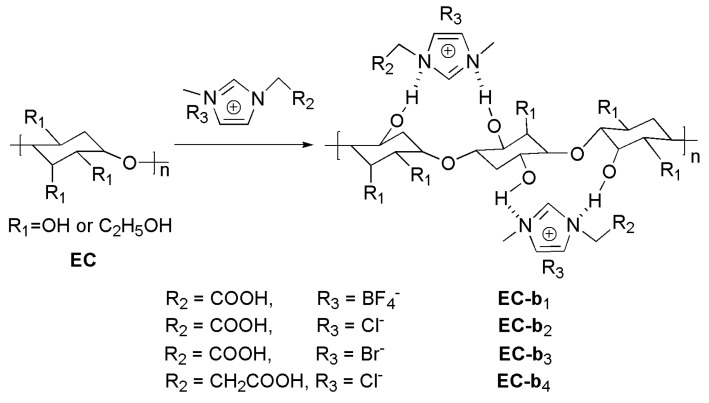
Preparation of EC blended with ionic liquids (EC-b1 - EC-b4).

Ethyl cellulose and ionic liquids were purchased from Chembee (Shanghai, China) and Greenchem ILs (Lanzhou, China), and were used as reactants. The ethyl cellulose we used was 45-55 MPa.s, 95% pure, and contained 5% toluene/isopropanol = 80:20. This is different from the purity and type of ethyl cellulose used in the literature, resulting in a certain deviation from the literature. Toluene and isopropanol are not plasticizers. Tetrahydrofuran was purchased from Kaitong Chemical Reagent Co. Ltd. (Tianjin, China) and was employed after distillation. EC grafted with ionic liquids (EC-g1–EC-g4 in Scheme 1) and EC blended with ionic liquids (EC-b1–EC-b4 in Scheme 2) were prepared according to Scheme 1 and Scheme 2. The details of the synthesis procedure and analytical analysis are presented in the following sections.

### 2.2. Preparation of Ethyl Cellulose Grafted with 1-Carboxymethyl-3-Methylimidazolium Tetrafluoroborate (EC-g1)

**Figure 1:**
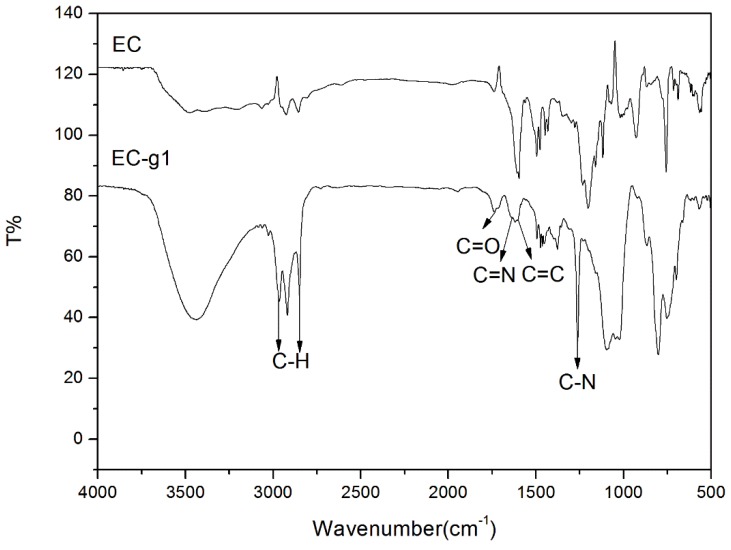
FT-IR spectrum of EC-g1 and EC.

Ethyl cellulose (1.45 g, 6.10 mmol), and pyridine (0.80 g, 10.12 mmol) were placed in a flask, evacuated for a 30 min, flushed with nitrogen and dissolved in THF (50 mL) at room temperature. Subsequently, 1-carboxymethyl-3-methylimidazolium tetrafluoroborate (1.20 g, 4.50 mmol) was injected dropwise, and stirred for 48 h at 65 °C. After the reaction mixture was cooled to room temperature, it was added to excess methanol (1000 mL) and precipitated for a week. The precipitate was concentrated by centrifugal separation and dried under vacuum to afford the desired product (1.02 g), obtained as a white solid, with a yield of 64.1%. IR (KBr, cm^−1^) bands were observed at 3080, 3020, 2960, 2930, 2860, 1760, 1640, 1620, 1500, 1470, 1400, 1380, 1348, 1250, 1100, 920, 890, and 620 (see Figure 1). The yield was calculated by this formula:

(1) $$\mathrm{Yield}=\frac{m_{s}}{\frac{m_{o}}{W_{u}}\times \left[d\times \left(W_{u}+W_{IL}\right)+\left(1-d\right)\times W_{u}\right]}\times 100\%,$$

where m_s_ is the mass of the grafted product obtained, m_o_ is the mass of EC added, W_u_ is the molecular weight of repetitive unit in EC, W_IL_ is the molecular weight of ionic liquid, d is the grafting degree in EC and d can be calculated from Equation (7).

### 2.3. Preparation of Ethyl Cellulose Blended Ionic Liquids (EC-b1– EC-b4)

According to Scheme 2, ethyl cellulose (1.45 g, 6.10 mmol) was added to anhydrous ethanol (50 mL) and stirred for 12 h at room temperature. Subsequently, ionic liquids (1.20 g, 4.50 mmol) were added to the solution and stirred for 12 h at room temperature.

### 2.4. Preparation of Gas Separation Membranes and Mechanical Testing

The dried products were dissolved in tetrahydrofuran at room temperature for 12 h. The uniformly mixed casting solution was carefully poured on a clean glass plate and was placed at room temperature for 24 h. The thicknesses of the films were measured by a thickness gauge. Thereafter, the solution was poured evenly on a clean glass plate to genedegree the membrane. The Spin Coater was used to prepare the membrane (the Spin Coater was purchased from Shanghai Sanyan Technology Co., Ltd., Shanghai, China; model SYSC-50). The casting membrane temperature was about 25 °C, the evaporation membrane temperature was about 25 °C, the evaporation time was 12 h, the casting thickness was about 0.5 mm and the relative humidity was 52%. The thicknesses of the membranes were measured by a thickness gauge (thickness gauge was purchased from Shanghai Liuling Instrument Factory, model was CH-1-B hand-type millimeter thickness gauge, the graduation value was 0.001mm, the measurement range was 0–1 mm and the error was about ≤0.001 mm). Mechanical properties were analyzed with a film tensile testing machine (XLW(PC)-500N, Sumspring, Jinan, China) at 25 °C.

### 2.5. Measurements and Calculations of the Gas Permeability Coefficient and the Separation Factor

The permeability coefficients of the mixed gases were measured by gas chromatographic method using the differential pressure gas transmission instrument (GTR-11MH type). The gas permeability coefficient P was calculated by the following, Equation (2) [27]:

(2) $$P=\frac{q\times K\times L}{a\times p\times t}\left(\mathrm{ml}•\mathrm{cm}•{\mathrm{cm}}^{-2}•s^{-1}•{\mathrm{cmHg}}^{-1}\right),$$

where, q is transmission volume (mL); K is auxiliary positive coefficient (the fixed value is 2), meaning that it is the setting point instrument by factory; L is film thickness (cm); p is permeability pressure (cmHg); t is measurement time (s); and a is the area of the gas permeation film (the fixed value is 0.785 cm^2^).

In this experiment, the gas separation factor was calculated by the Equation (3) [27]:

(3) $$\alpha_{{\mathrm{PCO}}_{2}/{\mathrm{PN}}_{2}}=\frac{P_{{\mathrm{CO}}_{2}}}{P_{N_{2}}},$$

(4) $$\alpha_{{\mathrm{PCO}}_{2}/{\mathrm{PCH}}_{4}}=\frac{P_{{\mathrm{CO}}_{2}}}{P_{{\mathrm{CH}}_{4}}},$$

where $P_{{\mathrm{CO}}_{2}}$, $P_{N_{2}}$ and $P_{{\mathrm{CH}}_{4}}$ can be calculated from Equation (2).

The diffusion coefficients (D) and the solubility coefficients (S) were calculated by the following equations [28,29]:

(5) $$D=\frac{L^{2}}{6T},$$

(6) $$S=\frac{P}{D},$$

where L and T are the amount of the thickness of the membrane and the time lag.

### 2.6. Analysis of the Grafting Degree in EC

The grafting degree is defined as the mass fraction of ionic liquid in EC. Using organic elemental analyzer, it was calculated by the following Equation (7) [30]:

(7) $$d=\frac{1}{2}N\%\times \frac{M_{\mathrm{IL}}}{M_{N}}\times 100\%,$$

where, d is the grafting degree in EC; N% is the mass fraction of N which was determined by using elemental analyzer. N content is expressed in wt%. M_IL_ is the molecular weight of ionic liquid and M_N_ is the molecular weight of nitrogen element.

## 3. Instruments

The gas permeability was measured at 25 °C using a differential pressure gas transmission instrument (GTR-11MH type, GTR TEC Corporation, Kyoto, Japan; the test area was 0.785 cm^2^. The instrument test temperature was 34 °C. And the test pressure was maintained at 49 KPa. The gas was a mixed gas, the content of the two components was the same, the test pressure was 0.1 MPa. The carrier gas was H_2_, and the pressure was 0.5 MPa). Infrared spectra were recorded on a Fourier transform infrared spectrometer (Spectrum Two, PE company, Waltham, MA, USA). The content of elemental N in grafted product was measured using an organic elemental analyzer (PE2400 SERIES II CHNS/O, PerkinElmer, Waltham, MA, USA).

## 4. Results and discussion

### 4.1. The Esterification of Ethyl Cellulose

Under appropriate reaction conditions, carboxyl groups in ionic liquid and hydroxyl groups in EC were reacted by esterification reaction (Scheme S1 in Supporting Information), which led to stable EC grafted with ionic liquids (Scheme 1, EC-g1–EC-g4).

Fourier transform infrared spectrometry (FTIR) was used to determine the molecular structure of the grafted ethyl cellulose. As shown in Figure 1, there is a strong peak at 1760 cm^−1^, which was attributed to C=O bond stretching vibration of ester group generate by grafting reaction between hydroxyl groups in EC and carboxyl groups in ionic liquid. The absorption peaks at 1620 cm^−1^ and 1640 cm^−1^ can be attributed to C=N and C=C bond stretching vibration of imidazole groups in ionic liquid, correspondingly. There are two weak spectral bands at 2960 cm^−1^ and 2860 cm^−1^, which were generated by the C–H bond stretching vibration of methyl and methylene groups in EC respectively. The C–H stretching vibrations in the imidazole-based double bond in the ionic liquid appear at 3080 cm^−1^ and 3020 cm^−1^, and its C–H bending vibration also appeared at 1400 cm^−1^. The C–N stretching vibrations of the imidazole group are observed at 1470 cm^−1^, 1250 cm^−1^ and 1348 cm^−1^. The other EC derivatives (EC-g2–EC-g4) also showed the similar IR spectra (Figure S1–S3 in Supporting Information). On the basis of the observed IR spectra, it was concluded that the EC grafted with ionic liquids were synthesized successfully.

The yields of the grafted products were calculated by weighing the products after drying (Table 1, EC-g1–EC-g4). The grafting degree of the final products was determined by N element content in the grafted products. The yields and grafting degrees of the products obtained in the experiment are shown in Table 1. The yields of EC grafted with ionic liquids were about 62.5–64.1% (Table 1, 2–5). As the EC derivatives were washed by an excess methanol, the yields were moderate, and pure products were obtained. The EC grafted with ionic liquids exhibited moderate grafting degrees (Table 1, 5.93–7.90%), which showed that the ionic liquids were successfully grafted into ethyl cellulose.

In this experiment, 1000 mL methanol solution was used to wash the grafted products for a week, which lost some unreacted ethyl cellulose to the methanol solution. That resulted in a decrease in the yield of the reaction.

EC is dissolved in solvent, but it is still an agglomerated macromolecule, and some hydroxyl groups are enclosed. The ionic liquid itself has a strong polarity, which affects the progress of grafting reaction. The above reasons can lead to low grafting degrees.

Through the GPC test, we found that the pure ethyl cellulose had an average molecular weight of 1.7 × 10^5^ and a molecular weight distribution of 2.68. The molecular weight of the grafted ethyl cellulose was lower than that of pure ethyl cellulose, and the molecular weight distribution was narrowed.

### 4.2. Gas Permeation Properties of Membranes

The EC and the modified EC were stirred and fully dissolved in tetrahydrofuran to achieve a uniform and transparent casting solution. The casting solutions were subsequently used to generate gas separation membranes with similar thicknesses. Overall, the materials exhibited good membrane forming abilities. In order to compare the effects of ionic liquids in the EC membranes on their carbon dioxide permselectivities, the gas separation performances for taking CO_2_ from CO_2_/N_2_ and CO_2_/CH_4_ mixtures were tested. It can be seen from Table 2 that the CO_2_/CH_4_ separation factors (12.0–18.8) in the membranes with EC grafted or blended with ionic liquids (Table 2, 2–9) were almost two times those of the pure EC membranes. However, the *P*CO_2_/*P*CH_4_ permselectivity values of silyl ethers of ethyl cellulose were observed to be in the range of 6.0–8.7 [19]. For a CO_2_/N_2_ gas mixture, the EC membranes modified by ionic liquids improved the separation factor to 31.9 from 20.6 for the pure EC membrane (Table 2). The *P*CO_2_/*P*N_2_ permselectivity values of silyl ethers of ethyl cellulose were observed to be in the range of 15–19 [19]. Thus, it can be confirmed that the imidazole ionic liquids are useful to improve the gas separation performance of the EC based membranes. This is because the imidazolium group in the ionic liquid interacts with CO_2_, which promotes the penetration of CO_2_ gas molecules and continues to undergo adsorption regulation within the membrane, ultimately resulting in high CO_2_ permeability. On the other hand, CH_4_ belongs to a regular tetrahedral structure and a non-polar molecule, but the ionic liquids added belong to a polar substance. According to the principle of similar compatibility, the solubility of methane in the membrane is poor, which causes a low permeation of CH_4_. Therefore there was a high CO_2_/CH_4_ selectivity [31].

The most important feature of the grafted EC membranes (Table 2, Nos. 2–5) is not only their high separation factor (12.0–18.8), but their excellent gas permeability coefficients (61.5–199 Barrer) for CO_2_/CH_4_ mixtures gas, which is better than pure EC. For instance, EC-g4 containing a flexible ethyl spacer in the ionic liquid exhibited the highest CO_2_ permeability coefficient of 199 Barrer with a separation factor of 12 for CO_2_/CH_4_ mixture gas. For an CO_2_/N_2_ mixture gas, EC-g4 also demonstrated the highest *P*CO_2_ value (Table 2, 194 Barrer). In our previous study [32], the flexible silyloxyl spacers in copoly (substituted acetylene) membranes were reported to be effective in enhancing their permeability coefficients. Therefore, it can be opined that the flexible ethyl spacer between imidazole and ester groups (EC-g4) largely governs the motion of imidazole, which strongly affects the CO_2_ permeability coefficient.

**Figure 2:**
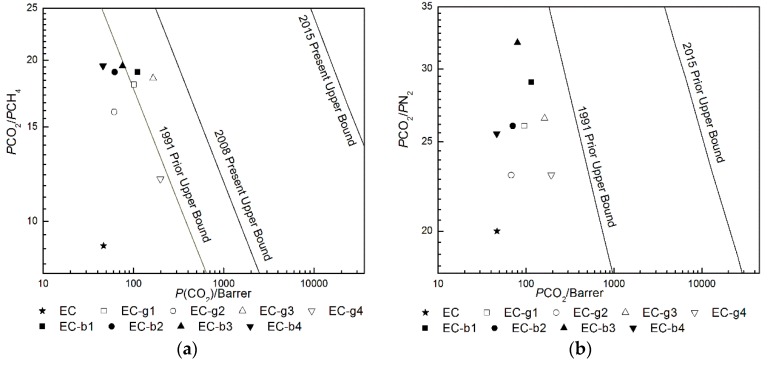
Plot of permselectivity vs permeability for the gas pairs (**a**) CO_2_/CH_4_ and (**b**) CO_2_/N_2_.

The EC-g3 derivative containing bromide exhibited the highest separation factor (18.8 Barrer) with a permeability coefficient of 165 Barrer (Table 2, No. 4), which was located above 1991 Robeson’s upper bound [17,33,34,35] and near to 2008 Robeson’s upper bound (Figure 2a). The separation performance of the gas separation membrane EC-g2 was lower than that of 1991 Prior Upper Bound; however, it was relatively close (Figure 2a). Overall, the gas permeation properties of the grafted EC membranes approximately displayed the following order: EC-g3 > EC-g1 > EC-g4 > EC-g2 > EC. It can be mentioned that halogen atoms are highly electronegative and have electron-absorbing induction effect, F > Cl > Br > I [36]. On the other hand, BF_4_^−^ has four fluorine boron bonds which are linked by SP_3_ hybrid orbital to form a stable tetrahedral molecule, so it has weak electron donating capacity. In this study, the electron donating capacities of anions were Br^−^ > Cl^−^ > BF_4_^−^. The free electrons on bromide can interact with Lewis acid CO_2_, which can make the imidazole group more stable. Therefore, the gas separation performance of the EC-g3 membrane containing bromide was observed to be the best.

On the other hand, EC and ionic liquids were also blended to develop composite films. There are pairs of lone electrons in the imidazole ring of the ionic liquid, which can form hydrogen bonds with hydrogen atoms on the hydroxyl group of ethyl cellulose. Due to the action of the hydrogen bonding, the ionic liquid and ethyl cellulose can interact firmly, forming relatively stable composite films. The EC and ionic liquid-blended membranes exhibited high separation factors (18.6–19.5, Table 2, Nos. 6–9) and low permeability coefficients (46.0–110 Barrer). As a large amount of ionic liquid is added to the EC membranes, fractional free volume may become small, resulting in low permeability coefficients [37]. However, it also causes high permselectivity for CO_2_. Because blending is a physical mixture without chemical reaction, the phase structure of blending membrane is two phasic. It can be observed from scanning electron microscopy (Figure S4 in Supporting Information) that some ionic liquids are agglomerated, so it is difficult to have a good interaction with CO_2_, resulting in low permeability coefficients. Grafting is a chemical reaction that can have a homogeneous phase structure. IL dispersed well in the EC, effectively promoting the permeability of CO_2_, thus, improves CO_2_ permeation efficiency. Based on the findings observed, it can be concluded that the ionic liquid improved the separation factor of CO_2_/CH_4_ significantly.

For the mixture of CO_2_ and CH_4_, the CO_2_ molecule as a whole has no polarity, but the O in CO_2_ has a unique pair of electrons, which can form a hydrogen bond with H in CH_4_ (see Scheme S2 in Supporting Information). When the mixed gas contacts the surface of the membrane, it may be adsorbed and dissolved on the surface of the membrane in the form of one molecule pair. In EC-g1, EC-g3 and EC-g4, SCO_2_ is larger in CO_2_/CH_4_ than those in CO_2_/N_2_ (See Table 2). In the diffusion process, the presence of ionic liquids broke the hydrogen bonds. The interaction between CO_2_ and IL makes CO_2_ permeate preferentially, which can achieve the purpose of separation.

CO_2_/N_2_ permeability of the modified EC membranes was also determined, and the results are shown in Table 2 and Figure 2b. CO_2_ permeability coefficients were observed to improve from 43.2 for pure EC membrane upto 194 Barrer for the modified EC membranes, indicating an improvement of 4.6 times over EC membrane. Among the grafted EC membranes (Table 2, numbers 2–5), EC-g3 containing bromide ion exhibited the highest CO_2_ permselectivity (*P*CO_2_/PN_2_ 26.2). EC-b3 also demonstrated the best *P*CO_2_/*P*N_2_ value of 31.9 among the blended EC membranes (Table 2, numbers 6–9). The separation performance of all gas separation membranes was observed to be under the 1991 prior upper bound (Figure 2b). Overall, the conclusion is similar to the case of CO_2_/CH_4_ mixture gas; i.e., bromide ion can enhance CO_2_ permselectivity (*P*CO_2_/*P*N_2_ or *P*CO_2_/*P*CH_4_). In other words, 1-carboxymethyl-3-methylimidazolium bromide was confirmed to be effective in enhancing the CO_2_ permselectivity of EC membranes.

### 4.3. Mechanical Properties of Polymer Membranes

The mechanical properties of the EC and modified EC membranes are shown in Table 3. Compared to EC, the elasticity moduli of the grafted membranes increased from 207 MPa for EC to 560–648 MPa; however, the blended membranes exhibited a decrease in modulus to 117–177 MPa. The results indicate that the grafted membranes (Table 3, numbers 2–5) are homogeneous, whose molecules are connected by covalent bonds. On the other hand, the blended membranes (Table 3, numbers 6–9) represent binary composites, which have weak physical connection, leading to reduced mechanical strength.

In the phenomenon shown in Table 3, we believe that in the blending system, the interior exists is two phases. EC is the continuous phase, the ionic liquid is the dispersed phase. The ionic liquid does have not much influence on the EC molecular structure. Although the content of ionic liquid is high, its caking property is weak, which has little effect on its mechanical properties. However, when EC is grafted with ionic liquid, the crystal structure of EC may change. The molecules are covalently bonded and intermolecular interactions are strong. With the different grafting degrees, the intermolecular forces will be different, showing different mechanical properties. The chains of ethyl cellulose were broken by grafting to decrease in molecular weight (See Table 1), which caused an increase in brittleness.

## 5. Conclusions

ECs grafted with ionic liquids were synthesized. Based on the infrared spectra, ionic liquids containing imidazole groups were successfully grafted into ethyl cellulose. The yields of ECs grafted with ionic liquids were about 62.5–64.1%, and the grafting degrees were 5.93–7.90%. The grafted products exhibited good membrane forming abilities. The CO_2_/CH_4_ separation factor in the membranes with EC grafted or blended with ionic liquids was almost two times compared to the pure EC membranes. Among these modified EC membranes, EC-g3 membrane was the best: the PCO_2_/PCH_4_ separation coefficient was 18.8 and the permeability coefficient (*P*CO_2_) was 199 Barrer. The bromide ion in EC-g3 can interact well with CO_2_, which promotes CO_2_ permeability. Thus, it can be confirmed that the imidazole ionic liquids are useful for improving the gas separation performances of the EC-based membranes.

## Figures and Tables

**Table 1:** Molecular weight, yield and grafting degree of pure ethyl cellulose and grafted with ethyl cellulose.

No.	EC Derivatives	Yield (%)	N-element Content (%)	Grafting Degree (%)	M_w_^a^(× 10^5^)	Mw/Mn^a^
1	EC	-	-	-	1.71	2.68
2	EC-g1	64.1	0.97	7.90	1.21	2.32
3	EC-g2	62.5	0.94	5.93	1.39	2.20
4	EC-g3	63.7	0.98	7.74	1.70	2.62
5	EC-g4	62.9	0.96	6.54	1.69	2.64

^a^ Determined by GPC correlating polystyrene standard with THF eluent.

**Table 2:** Gas permeation properties of EC membranes modified with ionic liquids.

NO.	Membrane	*P*CO_2_(Bar) ^a^ (*P*CO_2_/*P*CH_4_)	*D*CO_2_^b^ (*D*CO_2_/*D*CH_4_) ^c^	*S*CO_2_^d^ (*S*CO_2_/*S*CH_4_) ^e^	*P*CO_2_(Bar) ^a^ (*P*CO_2_/*P*N_2_)	*D*CO_2_^b^ (*D*CO_2_/*D*N_2_) ^c^	*S*CO_2_^d^ (*S*CO_2_/*S*N_2_) ^e^
1	EC	44.1 (9.01)	9.70 (0.21)	4.61 (42.9)	43.2 (20.6)	14.6 (1.82)	3.01 (11.8)
2	EC-g1	102 (17.8)	10.8 (0.07)	9.42 (262)	96.0 (25.9)	41.2 (1.31)	2.31 (20.5)
3	EC-g2	61.5 (15.8)	35.6 (0.07)	1.71 (228)	67.7 (22.6)	31.8 (1.41)	2.11 (15.9)
4	EC-g3	165 (18.8)	5.31 (0.08)	31.0 (246)	157 (26.2)	18.4 (0.73)	8.6 (38.7)
5	EC-g4	199 (12.0)	3.32 (0.08)	60.8 (147)	194 (22.5)	13.6 (0.71)	14.2 (30.7)
6	EC-b1	110 (18.6)	9.41 (0.91)	11.7 (20.0)	119 (25.8)	19.3 (2.21)	6.21 (11.7)
7	EC-b2	62.2 (18.8)	9.22 (0.71)	6.81 (26.7)	70.8 (26.2)	48.1 (2.21)	1.51 (11.7)
8	EC-b3	76.0 (19.5)	23.5 (0.92)	3.22 (21.9)	79.7 (31.9)	13.2 (3.71)	6.02 (8.71)
9	EC-b4	46.0 (19.2)	10.3 (0.81)	4.51 (23.8)	47.8 (23.9)	37.2 (2.22)	1.31 (11.1)

^a^ 1 barrer = 10^−10^ cm^3^(STP)*cm cm^−2^s^−1^cmHg^−1^, ^b^ In 10^−8^ cm^2^ s^−1^, ^c^ In 10^−2^, ^d^ In 10^−2^ cm^3^ (STP)*cm^-3^ cmHg^−1^, ^e^ In 10^2.^

**Table 3:** Mechanical properties of the membranes of EC modified with ionic liquids ^a.^

No.	Membrane	Thickness (mm)	Elongation (%)	Elasticity Modulus (MPa)
1	EC	0.1391	21.6	207
2	EC-g1	0.1448	4.22	648
3	EC-g2	0.1138	8.61	560
4	EC-g3	0.1591	3.92	644
5	EC-g4	0.1282	5.53	593
6	EC-b1	0.1338	16.2	117
7	EC-b2	0.0986	10.2	177
8	EC-b3	0.1158	8.71	119
9	EC-b4	0.0892	15.4	143

^a^ Tested at 5.00 mm/min speed. The standard spline had a length of 50 mm and a width of 10 mm.

## Supplementary Materials

The following are available online at https://www.mdpi.com/2073-4360/11/11/1900/s1, Scheme S1. Mechanism of pyridine-catalyzed esterification, Scheme S2. The coupling effect occurs of CO_2_ in the presence of CH_4_, Figure S1. FT-IR spectrum of EC-g2, Figure S2. FT-IR spectrum of EC-g3, Figure S3. FT-IR spectrum of EC-g4, Figure S4. SEM images of ethyl cellulose blended 1-carboxymethyl-3-methylimidazolium gas separation membrane.
