# Supplementary material for: High Efficiency Gas Permeability Membranes from Ethyl Cellulose Grafted with Ionic Liquids
Source: Polymers (Basel). 2019 Nov 18;11(11):1900. doi: 10.3390/polym11111900 (PMC6918432; doi:10.3390/polym11111900)
Supplement: Supplementary file 1 [file polymers-11-01900-s001.pdf]

Polymers **2019**

OPEN ACCESS

polymers

ISSN 2073-4360

www.mdpi.com/journal/polymers

Article

Supporting Information

High efficiency gas permeability membranes from ethyl cellulose grafted with ionic liquids

Jingyu Xu[1], Hongge Jia[1,*], Nan Yang[1,*], Qingji Wang[2], Guoxing Yang[3] , Mingyu Zhang[1], Shuangping Xu[1], Yu Zang[1], Liqun Ma[1], Pengfei Jiang[1], Hailiang Zhou[1], Honghan Wang[1]

[1] College of Materials Science and Engineering, heilongjiang province Key Laboratory of Polymeric Composition, college of architecture and civil engineering, Qiqihar University; Wenhua Street 42, Qiqihar, 161006, China.

[2] Daqing Oilfield Construction design and Research Institute, XiLing road 32, Daqing1637241, China.

[3] Daqing Petrochemical Research Center, Petrochemical Research Institute, China National Petroleum Corporation, Chengxiang Road 2, Daqing 163714, People’ s Republic of China.

* Corresponding author: Hongge Jia (Tel.: +86-452-2738752; E-mail address: jiahongge11@hotmail.com); Nan Yang (Tel.: +86-18204627375; E-mail address: 38281312@qq.com).

1.1 Mechanism of pyridine-catalyzed esterification

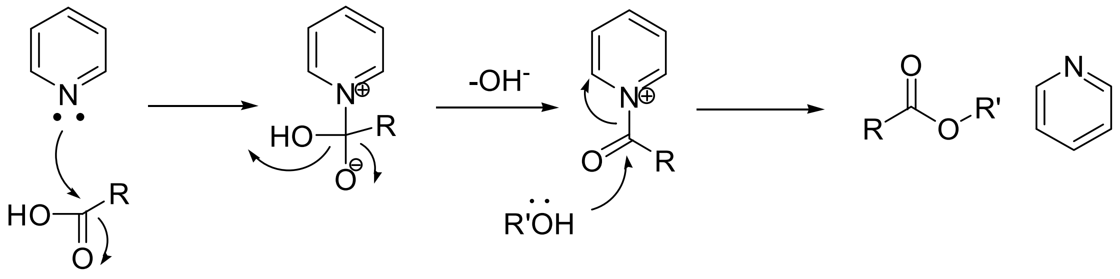

1 2 3 4

Scheme S1. Mechanism of pyridine-catalyzed esterification

The catalytic action of pyridine is shown in Scheme S1. We use pyridine as a catalyst. N on the pyridine contains a unique electron, which is electronegative and has an affinity. In the esterification reaction, the carbon on the carbonyl group is attacked by pyridine to form the intermediate 2. After 2 desorbed OH^- to form the 3, the oxygen of on the alcohol attacks the carbonyl carbon of 3 to form an ester. And the hydroxyl group removed from 2 combines with the free H^+ to form water. In a word, pyridine mainly catalyzes carbonyl carbon of carboxylic acids.

1.2 Preparation of ethyl cellulose grafted 1-carboxymethyl-3-methylimidazolium chloride (EC-g2)

EC-g2 was prepared by following the similar procedure as for EC-g1. The desired product (0.96 g) was obtained as a white solid with a yield of 62.5%. IR (KBr, cm^{-1}) bands were observed at 3160, 3120, 2960, 2930, 2860, 1740, 1680, 1620, 1480, 1400, 1350, 1250, 1120, 920, 800, 750 and 620（see SI, Fig. S1）.

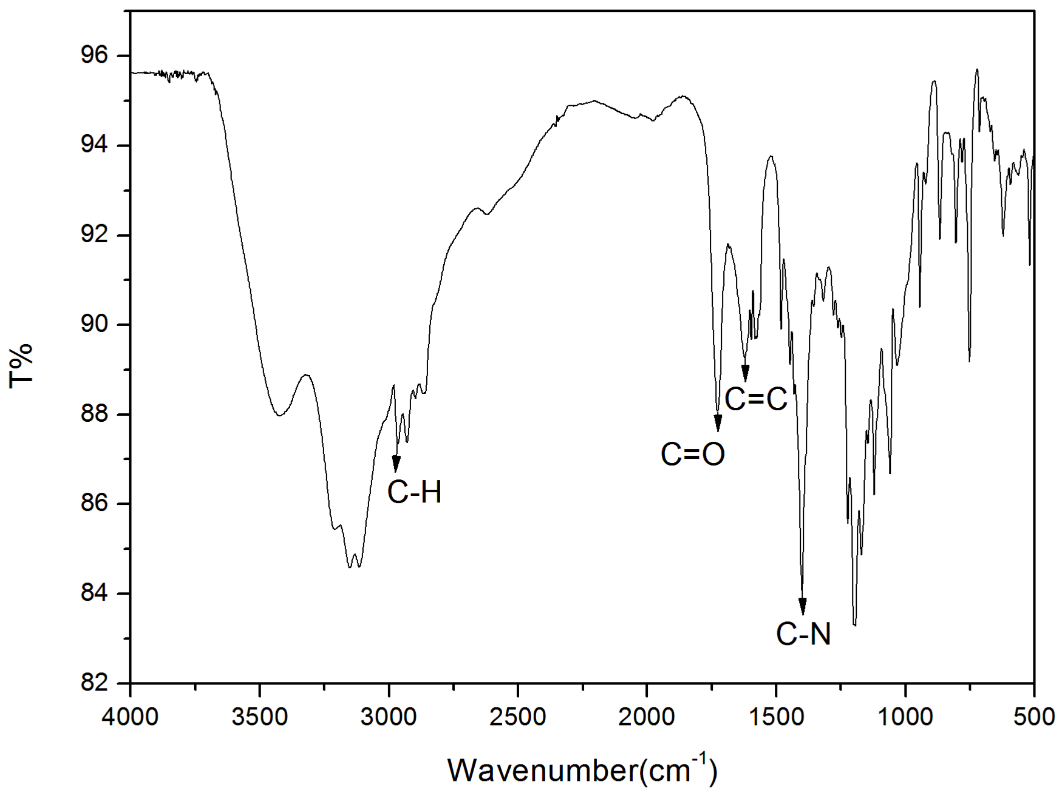

Figure S1. FT-IR spectrum of EC-g2

1.3 Preparation of ethyl cellulose grafted 1-carboxymethyl-3-methylimidazolium bromide (EC-g3)

EC-g3 was prepared by following the same procedure as for EC-g1. Similar to other case, the desired product (1.23 g) was obtained as a white solid with a yield of 63.7%. IR (KBr, cm^{-1}) bands were noted at 3160, 3120, 2930, 2900, 2860, 1740, 1640, 1580, 1440, 1400, 1348, 1260, 1090, 890, 780, 680 and 620 (see SI, Fig. S2).

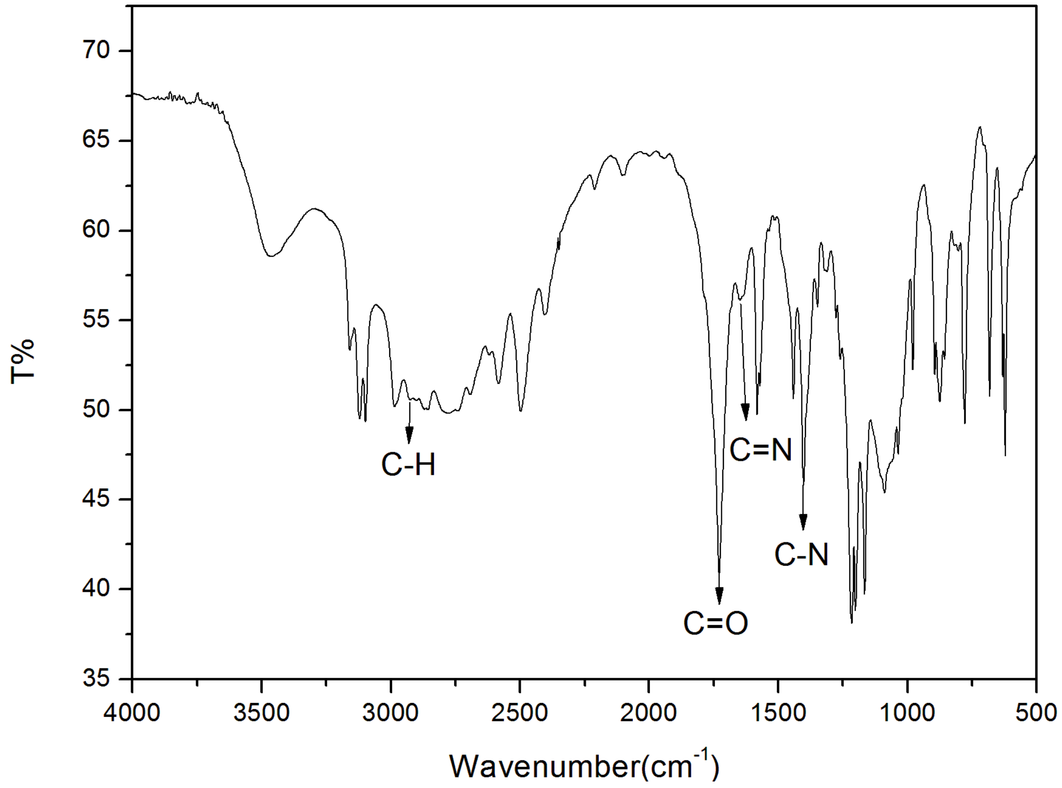

Figure S2. FT-IR spectrum of EC-g3

1.4 Preparation of ethyl cellulose grafted 1-carboxyethyl-3-methylimidazolium chloride (EC-g4)

EC-g4 was prepared by following the same procedure as for EC-g1. The obtained product (1.05 g) was a white solid, and the yield was 62.9%. IR (KBr, cm^{-1}) bands were observed at 2980, 2930, 2900, 1760, 1570, 1475, 1450, 1380, 1345, 1230, 1080, 1045, 900, 880, 805 and 620 (see SI, Fig. S3).

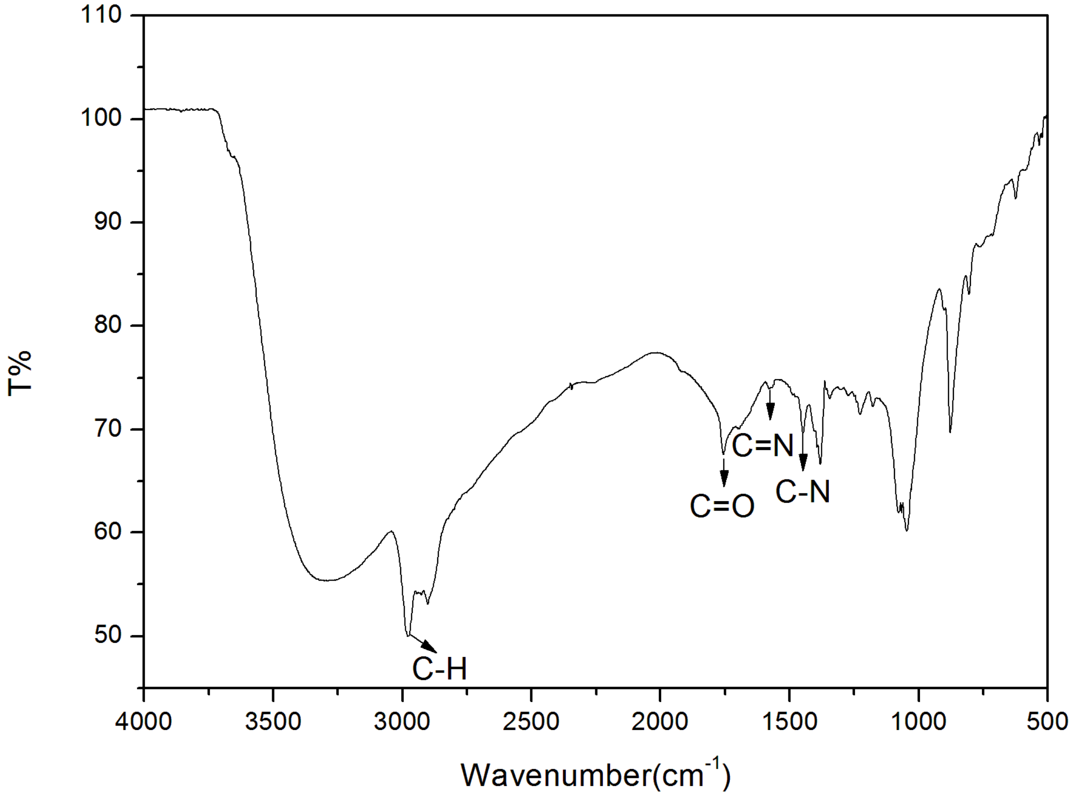

Figure S3. FT-IR spectrum of EC-g4

1.5 The scanning electron microscope of ethyl cellulose blended 1-carboxymethyl-3-methylimidazolium gas separation membrane

Some ionic liquid are agglomerated, so it is difficult to play a good interaction, thus, resulting in low permeability coefficients.

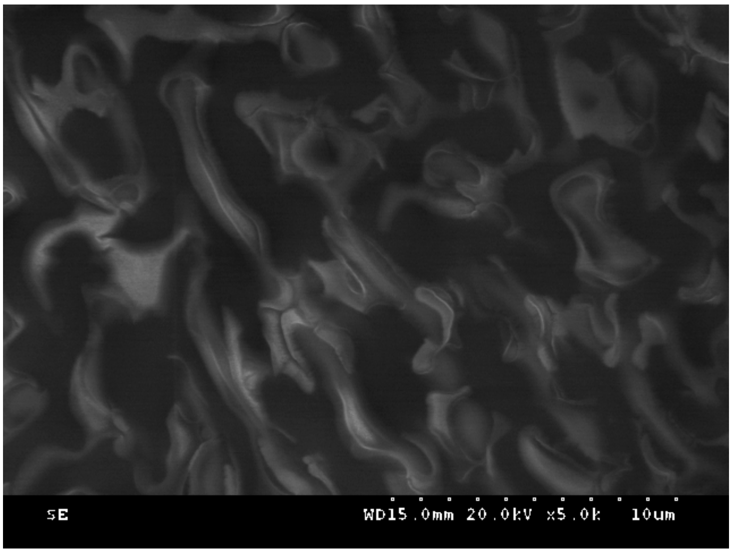

Figure S4. SEM images of ethyl cellulose blended 1-carboxymethyl-3-methylimidazolium gas separation membrane

The surface morphology of the synthesized material samples was observed by SEM (S-3400, Hitachi,Japan). And we added this into supporting information.

1.6 The coupling effect occurs of CO_2 in the presence of CH_4 and in the presence of N_2

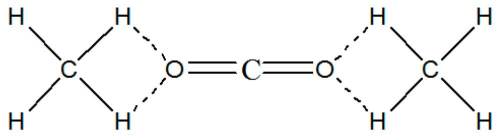

1

Scheme S2. The coupling effect occurs of CO_2 in the presence of CH_4

For the mixture of CO_2 and CH_4, the CO_2 molecule as a whole has no polarity, but the oxygen atom in CO_2 has a unique pair of electron, which can form a hydrogen bond with H in CH_4 (See Scheme S2). When the coupling gas contacts the surface of the membrane, it may be adsorbed and dissolved on the surface of the membrane in the form of 1 in Scheme S2. Therefore, in EC-g1, EC-g3, and EC-g4, S_{CO_2} were larger in CO_2/CH_4 than those in CO_2/N_2 (See Table 2). In the diffusion process, the presence of ionic liquids broke the hydrogen bonds. The interaction between CO_2 and IL makes CO_2 permeate preferentially, which can achieve good separation effect.
